# A Study of the Prognostic Factors for Late Cervical Lymph Node Metastasis and Distant Metastasis in Patients with cT1-2N0 Tongue Cancer

**DOI:** 10.3390/jcm13040976

**Published:** 2024-02-08

**Authors:** Fumitaka Obayashi, Koichi Koizumi, Nanako Ito, Mirai Higaki, Yasutaka Ishida, Atsuko Hamada, Sachiko Yamasaki, Ryouji Tani, Souichi Yanamoto

**Affiliations:** Department of Oral Oncology, Graduate School of Biomedical and Health Science, Hiroshima University, 1-2-3, Kasumi, Minami-ku, Hiroshima 734-8553, Japan

**Keywords:** cT1-2 tongue cancer, late cervical lymph node and distant metastases, tumor budding, prognostic factors, elective neck dissection

## Abstract

**Background:** Early-stage tongue cancer has a good prognosis in general; however, high-risk patients with late cervical lymph node and distant metastases have a poor prognosis. Elective neck dissection and postoperative chemoradiotherapy are considered for these patients, although no clear criteria have been identified for their evaluation. **Methods**: This retrospective observational study aimed to determine the predictive factors for late cervical lymph node and distant metastases in 102 patients with cT1-2N0 tongue cancer. The data regarding the demographic characteristics, as well as the depth of invasion, tumor budding, histological grade, and tumor–stromal ratio, among other things, were extracted from medical records. **Results**: We found that the potential lymph node metastasis rate was 27.5%. The significant clinical predictors of late cervical lymph node metastasis were the tumor thickness and endophytic growth pattern and the significant histopathological factors were poorly and moderately differentiated tumors and ≥3 tumor buds. In addition, the prognostic factors for distant metastasis included ≥4 lymph node metastases, ≥7 tumor budding, and moderate and poor tumor differentiation. **Conclusions:** The usefulness of tumor budding as a predictor of metastasis for tongue cancer was suggested. The findings of this study can help establish the criteria for evaluating the metastasis risk and prognosis of patients with tongue cancers.

## 1. Introduction

Patients with early-stage tongue cancer generally have a good prognosis; however, those who develop cervical lymph node or distant metastasis have a poor prognosis. It is estimated that 23–43% of patients with early-stage tongue cancer develop lymph node metastases [1]. Cervical lymph node metastasis decreases the survival rate to less than 50%; therefore, controlling cervical lymph node metastasis is essential to improving the survival of these patients. To date, there have been several discussions concerning elective neck dissection for oral cancer. Several prospective randomized trials have reported the superiority of primary resection combined with elective neck dissection (END) over resection alone [2,3]. However, approximately 70% of patients undergo cervical dissection in cases of negative cervical lymph metastases, and unnecessary cervical dissection should be avoided to reduce not only the risk of facial and accessory nerve paralysis, mastoid fistula, brachial neuropathy, and sympathetic nervous system dysfunction but also shoulder and neck strangulation and cosmetic concerns.

The eighth edition of the American Joint Committee on Cancer/the Union for International Cancer Control (UICC) TNM classification of oral cancer, revised in 2016, describes the concept of the “depth of invasion (DOI)” from the normal mucosa and recently reported that the DOI is a predictor of potential lymph node metastasis [4,5]. In addition, tumor budding (TB) [6,7], the pattern of invasion (POI) [8], and the tumor–stromal ratio (TSR) [9] have been reported to be associated with invasion patterns. However, there is no consensus regarding END for patients with N0-stage oral cancer. Furthermore, the definition of the high-risk group, in which cervical lymph node or distant metastasis is expected and a strict follow-up is recommended, is not yet clear, and the identification of high-risk factors for metastasis is needed. Therefore, in this study, we identified the clinical and pathological high-risk factors for late cervical lymph node metastasis (LCLM) and distant metastasis in patients with cT1–2N0 tongue cancer not treated with END and discussed the optimal cervical management based on a literature review.

## 2. Materials and Methods

### 2.1. Patients and Inclusion Criteria

This study was approved by the ethical review board of the Hiroshima University Clinical research Center (research approval no. E202300250/date 17 November 2023). A total of 102 patients with cT1–2N0 tongue cancer who visited our department between April 2011 and October 2021 with a postoperative follow-up period of at least 2 years were included in this study. The prognostic factors evaluated were extracted from the clinical medical records and retrospectively studied.

### 2.2. Treatment and Follow-Up

All patients were treated with partial tongue resection or radiotherapy, followed by a periodic follow-up. During the study period, patients with N0 tongue cancer underwent cervical dissection when the metastasis became apparent based on the “wait and see” policy. The TNM classification of oral cancer given in the seventh edition of the UICC classification was combined with that of the UICC eighth edition based on the medical records, and the tumor thickness of those with no clinical DOI was measured using magnetic resonance imaging (MRI) and computed tomography (CT). Monthly examinations were performed using ultrasound (US) every 1–2 months as well as CT and MRI every 3 months for 2 years postoperatively following the National Comprehensive Cancer Network (NCCN) guidelines.

### 2.3. Histopathological Assessment

- Pathological DOI

The pathological DOI (pDOI) was measured vertically from the straight line connecting the defined areas of the normal mucosa adjacent to the cancer lesion to the deepest part of the tumor according to the second edition of the Japanese Code of Conduct for the Treatment of Oral Cancer.

- Histological Grading

Histological differentiation was performed, and the tumors were sorted into two groups: a well-differentiated group and other; two cases of adenosquamous cell carcinoma (ASCC) were classified as other.

- Tumor Budding

TB is defined as the presence of a single tumor cell or a small tumor mass with fewer than five tumor cells isolated from the front of the invasion site, as previously reported [10]. The invasive front of the tumor on the specimen’s slide was observed at low magnification. The area with the most TB was observed with a 20× objective, and the area with the highest number of tumor buds was measured.

- Pattern of Invasion

The POI is also a pattern of tumor invasion of the tumor margins and is classified according to a scale of 1–5 [11]. We classified the POI into groups 1/2/3 and 4/5 and investigated the presence or absence of late lymph node metastasis and distant metastasis.

- Tumor–Stroma Ratio

The TSR was measured at low magnification, and the area with the greatest numbers of stromata and tumor cells present in the entire field of view was observed at high magnification and classified as follows: TSR ≥ 50% or TSR < 50% [12,13].

### 2.4. Statistical Analysis

All data were analyzed using JMP Pro 15 (JMP Statistical Discovery, Cary, NC, USA). Fisher’s exact test was used to compare groups, and the overall survival was estimated using the Kaplan–Meier method. The survival curves were analyzed using the log-rank test, and logistic regression analysis was used for multivariate analysis. The receiver operating characteristic (ROC) analysis revealed that the optimal cutoff value for the tumor thickness in LCLM was 4 mm (area under the curve: AUC = 0.72, specificity: 78.6%, sensitivity: 60.8%), the pDOI for LCLM was 2 mm (AUC = 0.72, specificity: 52.2%, sensitivity: 93.3%), the cutoff value for number of lymph node metastasis for distant metastasis was 4 (AUC = 0.75, specificity: 54.1%, sensitivity: 71.4%), and the pDOI for distant metastasis was 4.25 mm (AUC = 0.61, specificity: 71.4%, sensitivity: 60.0%).

### 2.5. Clinical and Pathological Items Collected from Medical Records

Study 1: We conducted a clinical study of the prognostic factors for late cervical lymph node and distant metastases in patients with cT1–2N0 tongue cancer. Various clinical parameters such as age, sex, T-stage, tumor size, tumor thickness, the presence and timing of lymph node metastasis and distant metastasis, the cervical lymph node metastatic area, the number of metastatic lymph nodes, prognosis, extranodal extension (ENE), and postoperative adjuvant treatment were assessed.

Study 2: We conducted a clinicopathological investigation of the prognostic factors for late cervical lymph node and distant metastases in patients with resected pT1–2 tongue cancer. A total of 61 primary cases of cT1–2 tongue cancer treated with surgical resection and detailed pathological information were included. Clinical parameters, such as ENE and postoperative adjuvant treatment, and pathological parameters, such as the degree of differentiation, mode of invasion (TB, POI, and TSR), neuro-vascular invasion, and pDOI, were examined as prognostic factors for LCLM and distant metastases.

## 3. Results

### 3.1. Baseline Characteristics of the Patients

**Figure 1:**
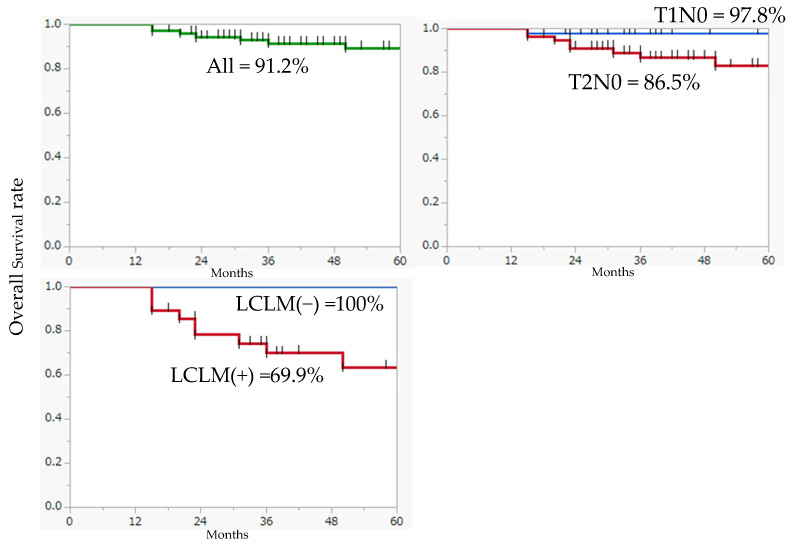
The 3-year overall survival rate for all patients included in this study.

The median age of the 102 patients (54 males and 48 females) was 66 years (range, 23–94 years). T1 and T2 tumors were observed in 45 and 57 patients, respectively. Overall, 74 patients underwent surgical treatment, and 28 patients received radiotherapy. Among those who received radiotherapy, 11 and 16 patients received Au grains and Ir-192 radiotherapy, respectively (Table 1). LCLM was observed in 28 patients, and the incidence of potential lymph node metastasis was 27.5%. Distant metastases were observed in seven patients (6.9%), and eight patients died of the primary disease. The 3-year overall survival rate for all patients included in this study was 91.2% (97.8% for T1 tumors and 86.5% for T2 tumors) and 69.9% for those with cervical lymph node metastases, indicating a poor prognosis (Figure 1). Cases of inoperability were not observed owing to the expansion of LCLM.

### 3.2. Study 1

In the univariate analysis, the predictive factors for postoperative cervical lymph node metastasis, including the tumor stage classification, a length, diameter, and thickness greater than 4 mm and the endophytic growth patterns, were significantly associated with the prognosis. Multivariate analysis revealed that a tumor thickness greater than 4 mm and an endophytic growth pattern were independent poor prognostic factors (Table 2).

**Figure 2:**
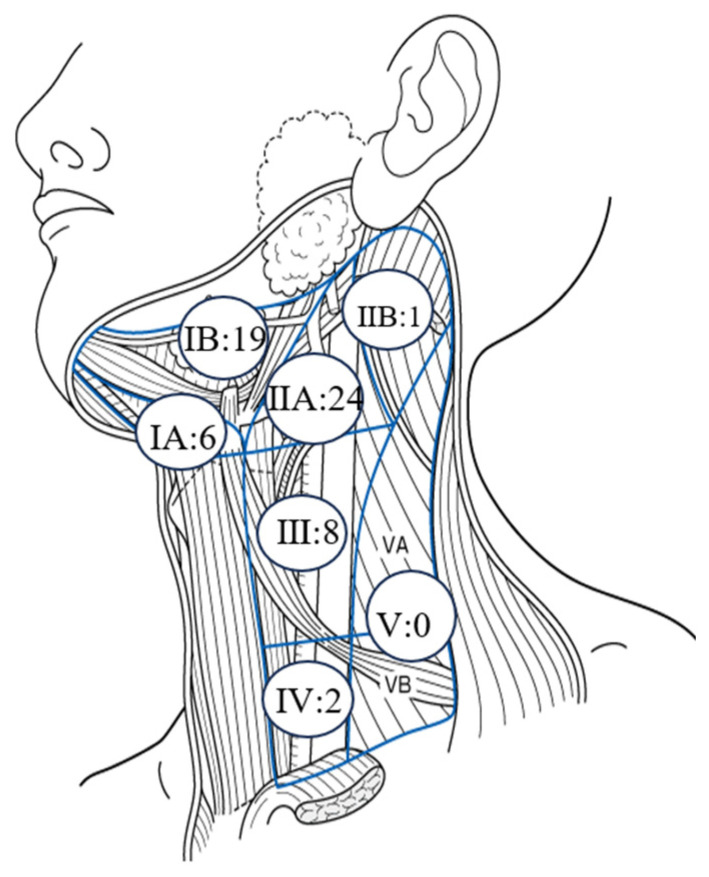
Number of metastatic cervical lymph nodes.

Table 3 presents the metastatic status and prognosis of 28 patients with LCLM. Lymph node metastases were concentrated at levels I–III; however, there were two cases of metastasis to level IV and no metastasis to level V (Figure 2). There was no metastasis to the lingual lymph nodes, including the para-hyoid lymph node. A total of 15 patients underwent radical neck dissection (RND), 7 underwent supraomohyoid neck dissection (SOHND), and 6 underwent extended SOHND (ExSOHND). Four or more lymph node metastases were observed in five patients. In all patients, postoperative lymph node metastasis occurred within 1 year, and, notably, 21 patients experienced ENE. Most patients received chemoradiotherapy as postoperative adjuvant therapy. The local and cervical lymph node metastasis control was considered good in most patients; however, seven patients developed distant metastasis after cervical dissection and died from distant metastasis.

Distant metastases occurred within 18 months after cervical neck dissection, and the distant metastatic sites were the lungs, mediastinum, and bones. Analysis of the prognostic factors for distant metastasis was performed in 28 patients with lymph node metastasis. The results suggested that the presence of four or more lymph node metastases was a prognostic factor for distant metastasis (Table 4).

### 3.3. Study 2

**Figure 3:**
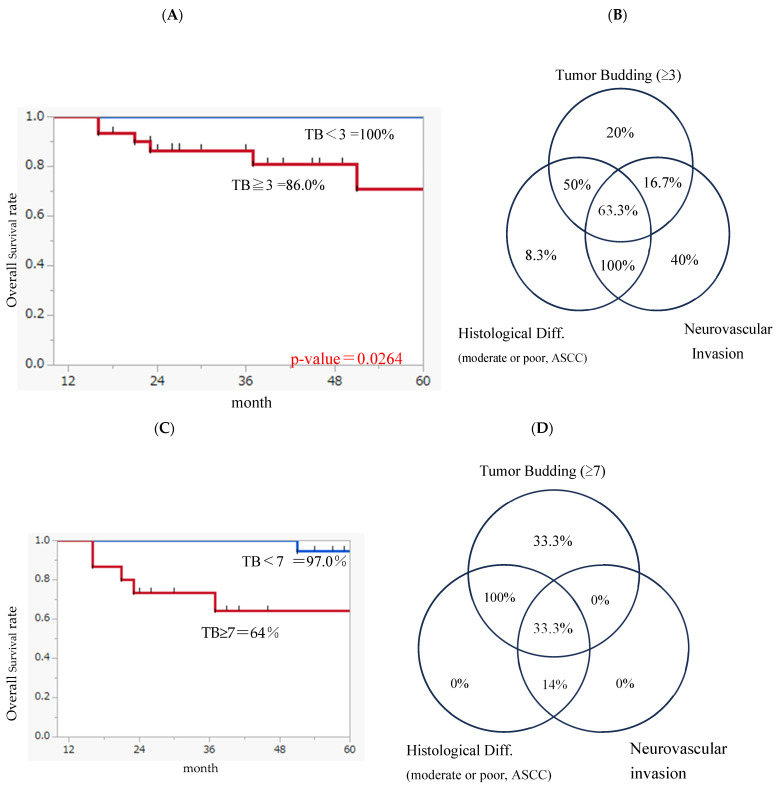
(**A**) Kaplan–Meier overall survival (tumor buds < 3 vs. tumor buds ≥ 3); (**B**) relationships between neurovascular invasion, histological differentiation, and TB for LCNM, (**C**) Kaplan–Meier overall survival (tumor buds < 7 vs. tumor buds ≥ 7), (**D**) Relationships between neurovascular invasion, histological differentiation, and TB for distant metastasis.

Next, the pathologic characteristics of 61 surgical cases of cT1-2N0 tongue cancer were examined to identify the clinicopathologic prognostic factors for LCLM. In the univariate analysis, the histological differentiation grade, the pDOI, three or more tumor buds, and neurovascular invasion were significantly associated with late cervical lymph node metastasis. In the multivariate analysis, histological differentiation grade and TB were considered independent predictive factors of LCLM (Table 5A). The survival analysis showed a significantly lower 3-year survival rate in the tumor buds ≥ 3 group than in the tumor buds < 3 group (Figure 3A). TSR and pDOI, which are related to the mode of invasion, showed a significant correlation with the number of tumor buds, whereas the pDOI showed a weak correlation with the tumor buds. Similarly, the histological differentiation grade and the presence of >7 tumor buds/neurovascular invasions were identified as independent prognostic factors for distant metastasis (Table 5B).

The survival analysis showed that the 3-year survival rate significantly decreased in the tumor buds ≥ 7 group compared with that in the tumor buds < 7 group (Figure 3C). When the relationships between neurovascular invasion, histological differentiation, TB, and tumor thickness were examined, cases with LCLM and distant metastasis tended to be concentrated in areas where each category overlapped (Figure 3B,D).

## 4. Discussion

The tongue has a complex three-dimensional structure. It is capable of multidirectional contraction and deformation and is characterized by an abundant supply of lymphatic vessels and neurovascular bundles. These anatomical features allow for the multidirectional invasive metastasis of tongue cancer. Furthermore, tongue cancer demonstrates epidemiological features with a high incidence frequency and is considered an atypical subtype among oral cancers.

Tongue cancer is more likely to metastasize to the cervical lymph nodes, and the NCCN guidelines for oral cancer suggest that END should be considered depending on the DOI. Weiss et al. [14] reported that END should be performed if the potential lymph node metastases is 20% or higher; however, even in Japan, most facilities have potential lymph node metastases of higher than 20%, and Okura et al. reported a cutoff value of 44.4% for lymph node metastasis using a similar approach [15]. Although there has been a considerable amount of debate on the advantages and disadvantages of prophylactic neck dissection, a consensus has not been established, and different institutions have different attitudes toward the treatment of metastatic disease. Sentinel node biopsy (SNB), which has emerged as a compromise between these two treatment strategies, END and the “wait and see” policy, is a less invasive treatment based on the SNB of breast cancer and is now gaining interest in the treatment of early-stage tongue cancer. A positive sentinel lymph node requires complete cervical dissection. Furthermore, the cost-effectiveness is high in patients with a negative SNB; however, a significant increase in costs is observed in those with a positive SNB. Although the true pN can be determined, a high false negative rate is noted in some subtypes of tongue cancer [16,17,18], and the provision of adjuvant therapy may be delayed because a second cervical neck dissection is required. Therefore, SNB should be limited to cases that are unlikely to develop lymph node metastasis and is unlikely to be indicated for thick tongue or floor of the mouth cancers. The NCCN guidelines state that SNB is indicated only for thin oral squamous cell carcinomas of up to 2 mm. In addition, radio isotope facilities need to be established and are not yet available for medical insurance coverage in Japan; therefore, this is not common practice.

Although END was not performed during the study period, the potential lymph node metastasis rate was 27.5%, which is consistent with that reported in previous studies. The 3-year overall survival and disease-specific survival rates were similar (91%), and the regional control rate was 85%. Compared with the results of previous studies [1,2,15], neck dissection for late lymph node metastases showed a better prognosis., and the data did not significantly differ from those reported in previous studies [19] of END. However, in the present study, 75% of patients with LCLM experienced ENE, with a higher ENE rate than that reported in the previous literature (30–71%) [20,21]. The purpose of performing END is to accurately identify and predict the prognosis of cervical lymph nodes, remove metastatic lymph nodes that cannot be detected using imaging modalities, and determine the postoperative treatment prognosis. A meta-analysis of the sensitivities of CT, MRI, and US in patients with clinically N0 head and neck cancer reported sensitivity rates of 47.0%, 56.6%, and 63.3% for CT, MRI, and US, respectively [22]. Despite the current improvements in imaging accuracy, the accuracy of the combined results of different imaging modalities is approximately 70–80%, and 1–3 mm micrometastases cannot be identified. If END is considered part of an examination that cannot be substituted for diagnostic imaging, its validity is assured. In this study, seven cases of distant metastasis were preceded by LCLM, and cervical lymph node metastasis was a major factor in the decreased survival rate. We believe that a further improvement in survival can be expected if END is performed in patients who are considered to be at a high risk of metastasis before cervical lymph node metastasis becomes apparent. Therefore, in general, END should be considered for patients at high risk of metastasis, those for whom performing regular cervical follow-ups is difficult, and those with tumor subsites such as the floor of the tongue and mouth.

Tumor thickness and DOI are not synonymous. Tumor thickness is the distance from the deepest part of the tumor to the mucosal surface, and the DOI is the distance from the newest part of the tumor to the basement membrane of the adjacent normal mucosa. Previously, oral cancer metastasis was associated with tumor thickness [23,24]. Furthermore, previous studies have reported that the cutoff value for metastasis varies by site, with a cutoff of 4–5 mm for the tongue and 1.5 mm for the floor of the mouth [25,26,27,28,29,30]. Recently, the DOI of a tumor has been reported to reflect the tumor invasiveness more accurately than the tumor thickness. In the present study, the multivariate analysis suggested that a clinically significant inward growth and a tumor thickness of 4 mm or higher are independent risk factors for distant metastasis, similar to those reported by Shinbashi et al. [31]. In contrast, there were no significant differences in the tumor length, diameter, or stage classification, suggesting that the vertical depth from the mucosal surface is important. However, as reported by Iandelli et al., potential lymph node metastases are not more common in patients with T2 tumors than in those with T1 tumors [32], and the current T-stage classification may not clearly distinguish between the indications for END. Risk classification of potential lymph node metastasis in combination with histologic differentiation as well as tumor depth has also been reported [33,34], although it is not limited to tongue cancer. Therefore, it may be necessary to consider histological differentiation and other factors when deciding whether to perform END. Furthermore, some tongue cancers that were thin but located near the border of the base of the tongue showed LCLM and distant metastasis, suggesting that metastasis may differ by site, even if tongue cancer is present at the same anatomical location.

Regarding the extent of prophylactic neck dissection, in patients with cN0 tongue cancer, Pantvaidya et al. [35] reported a 91% pickup rate for level I–III dissection, and the addition of level IV dissection increases the pickup rate to 96%. Studies have also reported that level IIB metastasis may be encountered if level IIA is positive. The rate of late metastasis to level IV is reported to be only 6–8% [36], and the skip metastasis rate to levels III and IV is also low [37]; in the present study, there were two cases of metastasis to level IV (1.9%) and no metastasis to level V. A randomized comparison of modified RND and SOHND reported no difference in survival [38], and SOHND is considered appropriate for prophylactic dissection, which has been performed in many healthcare centers. Lindberg et al. [39] reported that 6–12% of tongue cancers have contralateral metastases depending on the proximity to the midline of the tumor, but no contralateral metastasis was observed in this study. This may be because the patients had early-stage tongue cancer, and none of the tumors were highly or deeply invasive. In addition, the only case of metastasis to the IIB region in this study was a case in which TB revealed high levels of neurovascular invasion and distant metastasis, leading to death, despite the presence of a superficial carcinoma with a pDOI of 1.5 mm. However, IIB-sparing neck dissection may compensate for incomplete IIA resection and should be performed only after adequate training, although it has not yet become the standard of care [40,41]. A higher frequency of metastasis in the IIB region has been reported for tongue cancer (especially posteriorly) compared with other subsites [36]; therefore, a greater oncological advantage can be obtained by dissecting level IIB than considering the risk of accessory nerve injury.

Among the 28 patients with cervical metastases, those with more than four metastases were significantly more likely to have distant metastases. Of the eight patients with distant metastases, all but one refused chemoradiotherapy (CRT); however, the current postoperative CRT regimen with high-dose cisplatin does not contribute to distant control [42], and strong adjuvant therapy should be introduced.

The histopathological examination and statistical analysis of cervical lymph node and distant metastases revealed the utility of TB, which can be easily measured quantitatively and may be a useful predictive factor. TB is the result of the loss of cell adhesion and enhanced invasive capacity, which may imply the aggressiveness and diffuseness of the malignancy. Xie et al. reported the usefulness of the International Tumor Budding Consensus Conference (ITBCC) 2016 TB scoring method for tongue cancer, and several retrospective studies on TB have been conducted to date [43]. The cutoff values for tumor buds in oral squamous cell carcinoma vary from 3 [44] to 10 [45] units, while some studies have reported 4 or 5 units in early-stage tongue cancer; therefore, no consensus has been established.

In this study, tumors with three or more budding events (moderate budding) at the invasive tip were more likely to develop cervical metastases; specifically, tumors with seven or more budding events (severe budding) were associated with a significantly decreased survival rate. In addition, the TSR and POI were correlated with TB, and both factors were considered useful in the univariate analysis, although they were not used in the multivariate analysis.

Furthermore, the pDOI showed a weak correlation with TB. TB may describe the correlation between tumor progression and prognosis. Currently, the cutoff values of the pDOI for cervical lymph node metastases vary from 3 mm [46] to 6 [47] mm. Because the specimen contraction rate at the time of pathological specimen preparation differs among institutions [48,49], establishing a standard is difficult. In this study, we found that the mean clinical tumor thickness was 3.9 mm, but the mean pathological tumor thickness of the resected tumors (n = 61) was 3.4 mm, indicating a mean difference of approximately 13% between the clinical and pathological findings. The cutoff value of the pDOI was calculated to be approximately 2 mm based on the ROC analysis. However, pDOI was not an independent predictor of cervical lymph node metastasis in the multivariate analysis. Although the number of cases in which WPOI-5 was detected in this study was small and thus unreliable for analysis, several false negative cases were likely noted because WPOI-5 could not be detected unless there were distant tumor masses in the field of view of the specimen.

In summary, it may be difficult to predict cervical lymph node or distant metastasis using only one category, such as the pDOI or TB. When a Venn diagram was used to represent the aggregation relationship, the greatest percentage of lymph node metastases was clustered in the overlapping areas. The combined use of the tumor depth, tumor–host interaction such as TB, the POI and TSR, the degree of differentiation, and neurovascular invasion was considered to further increase their usefulness as predictors. Among 28 and 74 patients who received radiotherapy and underwent surgery as the primary treatment, the biopsy specimen did not provide sufficiently detailed information regarding the histological differentiation, mode of invasion, and vascular and nerve invasion. Therefore, it is necessary to establish a method that can predict the prognosis using biopsy specimens.

A prospective observational study of prophylactic neck dissection for cN0 cancers (END-TC Study: UMIN000027875) [50] is currently underway in Japan, and together with a randomized phase III study (RESPOND: JCOG1601) [51] comparing partial tongue resection plus neck dissection with partial tongue resection alone for stage I/II tongue cancer, the criteria for the prognostic predictors of END and their usefulness will be clarified.

### Study Limitations

The NCCN guidelines list perineural invasion, vascular invasion, and lymphatic invasion as different risk factors for recurrence and metastasis, but the older data in this study did not separate them and could not be analyzed separately.

In addition, this is a single-center retrospective study involving a small number of patients, and several biases may exist. Further studies with larger sample sizes are required to validate these findings.

## 5. Conclusions

In this study, we found that, clinically, endophytic proliferation and a tumor thickness of 4 mm or greater and, histopathologically, ≥3 tumor buds, high-grade histologic differentiation, and neurovascular invasion are the high-risk factors for distant metastasis. In patients with these high-risk factors, END with tongue resection may further improve the survival rates. However, as this was a single-center retrospective study, more studies are needed to validate these findings.

## Figures and Tables

**Table 1:** Details of included cases (cT1-2N0 tongue cancer n = 102).

		Cases	(%)
age		23~94 y	
	mean	66 y	
sex	M:F	54:48	
T-Stage	T1	45	44.1
	T2	57	55.9
Treatment	Surgery	74	72.5
	Radiotherapy	28	27.5
	Au	11	
	Ir	16	
	external irradiation	1	
LCLM	No	74	72.5
	Yes	28	27.5
Distant metastasis	No	95	93.1
	Yes	7	6.9

**Table 2:** Statistical analysis for LCLM.

	Univariate			Multivariate		
	LCLM					
	No	Yes	*p*-Value	HR	95%CI	*p*-Value
Age			0.4618			
<65	34	15				
≥65	43	13				
sex			0.379	1.15	0.41–3.27	0.78
Male	37	17				
Female	37	11				
T-Stage			0.0247 *	1.8	0.30–13.8	0.51
T1	38	7				
T2	36	21				
Tumor Size			0.0076 *	0.29	0.046–1.58	0.157
<20 mm	41	7				
≥20 mm	33	21				
Tumor Thickness			0.0007 **	0.284	0.077–0.94	0.0399 *
<4 mm	45	6				
≥4 mm	29	22				
Tumor growth pattern			0.0015 **	0.3313	0.117–0.96	0.044 *
Superficial/Exophytic	65	15				
Endophytic	11	13				

* *p* < 0.05, ** *p* < 0.005.

**Table 3:** Status of lymph node metastasis and prognosis (n = 28).

Method of Neck Dissection	RND	15	Number of metastatic lymph nodes	1	13
	ExSOHND	6		2	5
	SOHND	7		3	5
				4~	5
Region of LCLM	I	5	adjuvant treatment	Non	7
	I, II	10		Chemo therapy	0
	I–III	11		Radiotherapy	0
	I–IV	2		Chemoradiotherapy	21
	I–V	0	Distant metastasis	Non	0
Duration until lymph node metastasis appears	~3 months	11		Yes	7
	4~6 months	12	Prognosis	Alive	20
	7~9 months	5		Cause specific death	8
	10~12 months	0		by local recurrence	1
	12 months~	0		by regional recurrence	0
ENE	No	7		by distant metastasis	7
	Yes	21			

**Table 4:** A statistical study of prognostic factors for distant metastasis in patients with tongue cancer after neck dissection (n = 28).

	Distant Metastasis		
	No	Yes	*p*-Value
Number of metastatic lymph nodes			0.0012 **
<4	20	2	
≥4	1	5	
ENE			
No	7	0	0.14
Yes	14	7	
Region of LCLM			
~II	13	2	0.19
~III/~IV	8	5	
Post-operative treatment			
Non	6	1	0.64
Adjuvant CRT	15	6	

* *p* < 0.05, ** *p* < 0.005.

**Table 5:** (**A**) A statistical study of pathologic prognostic factors for LCLM in patients with early-stage tongue cancer. (n = 61). (**B**) A statistical study of pathologic prognostic factors for distant metastasis in patients with early-stage tongue cancer (n = 61).

(A)							
	Univariate			Multivariate			
	LCLM		*p*-Value	HR	95%CI	*p*-Value	
	No	Yes					
Histological differentiation			0.015 *	0.19	0.034–0.894	0.035 *	
well	30	4					
moderate/poor/ASCC	16	11					
pDOI			0.0083 **				
<2 mm	24	2					
≥2 mm	22	13					
Tumor Budding			<0.0001 **	6.99 × 10^−9^	0.0–0.12	<0.0002 **	
<3	31	0					
≥3	15	15					
TSR			0.0031 *				
<50%	36	5					
≥50%	10	10					
Neuro-vascular invasion			0.0018 **	0.62	0.09–3.50	0.59	
Neither of the above	34	4					
Any of the above	12	11					
POI			0.014 *	1.11	0.94–15.8	0.94	
1,2,3	24	2					
4,5	22	13					
**(B** **)**							
	**Univariate**			**Multivariate**			
	**Distant Metastasis**		***p*** **-Value**	**HR**	**95%CI**		***p*** **-Value**
	**No**	**Yes**					
Histological differentiation			0.1606	4.27 × 10^−8^	0–0.467		0.0129
well	33	1					
moderate/poor/ASCC	23	4					
pDOI			0.17				
<4.25 mm	40	2					
≥4.25 mm	16	3					
Tumor Budding			0.0005 **	1.17 × 10^−15^	0–0.041		<0.0001 **
<7	46	0					
≥7	10	5					
TSR			0.0026 **				
<50%	41	0					
≥50%	15	5					
Neuro-vascular invasion			1.00	5.7 × 10^−8^	0.0005–0.643		0.025
Neither of the above	35	3					
Any of the above	21	2					
POI			0.065				
1,2,3	26	0					
4,5	30	5					

* *p* < 0.05, ** *p* < 0.005.

## Data Availability

All raw data used in the current study can be found in the archives of Hiroshima University, the Department of Oral Oncology.
